# Report of a Case of Narcotism from the Exhibition of Sulphate of Morphia

**Published:** 1846-01

**Authors:** Thomas Carroll

**Affiliations:** Cincinnati, Ohio


**Art. II.**—*Report of a Case of Narcotism from the Exhibition of Sulphate of Morphia.* By Thomas Carroll, M. D., of Cincinnati, O.

Mr. B., aged 47 years, had been intemperate during 20 years of his life, but had reformed two years before the occurrence about to be related. In July, 1845, he arrived in Cincinnati, and a few days afterwards was taken ill with remittent fever, which, however, was not of a serious character. Dr. H. prescribed for him. The disease had yielded so far that the patient was sitting up, when the Doctor concluded that tonics would be useful, and wrote the following prescription, as he thought:

℞ Sulph. quinine, grs. xxiv.
Hydrargyri sub., grs. xxxvi.
M. Make into xij. powders.

But instead of the sulphate of quinine, he wrote sulphate of morphia. The powders were put up according to the prescription, and one was directed every two hours. The first dose was taken about 9 o'clock, A.M., on the 11th of July. The doctorr saw the patient at 1 P.M., after three doses had been given, he was then sitting up, but quite sleepy. This symptom made the doctor think that congestive fever was occurring, and he ordered, as the best preventive, a continuance of the medicine. The patient was again seen by him at 7 o'clock, P.M., when he was in a comatose state which led the doctor to the conclusion that a bad case of congestive fever had sure enough set in. He directed that the remaining six powders (for the patient had already taken six) should be sent back to the apothecary, and two grains of quinine added to each. This was done, and the medicine was continued, until three doses more were taken. The last dose administered, was got into his mouth by forcing the jaws apart with an instrument; but when the time rolled round for another dose, the masticatory muscles were so tight that the mouth could not be opened even by force. Alarm now seizied on the friends of the patient, and as the Doctor's health would not allow him to go out in the night, I was called in. When I arrived, I found, by inquiry, that the patient had been bathed in a cold sweat for sometime before the doctor had seen him at 7 o'clock, and that stertorous breathing had existed for hours. I found the extremities completely paralyzed, and bathed with cold perspiration; his breathing was laborious and irregular, sometimes amounting almost to a complete cessation. His masticatory muscles were so firmly fixed that the jaws could not be forced open; and he had not swallowed anything since the exhibition of the last dose of medicine.

I became satisfied that opium had been given in some form or other, and, therefore, went to the apothecary and examined the prescription. No sulphate of quinine was directed in it, but 24 grains of the sulphate of morphia had supplied the place; and it was certain that a mistake had been made in writing the prescription.

Eighteen grains of sulphate of morphia had now been given within 16 hours; and but two hours had elapsed from the exhibition of the last dose, until I saw the patient. I desired consultation, and Dr. Lawson was called in. We determined on the cold douche over the head and body, which we directed to be repeated every half hour; and that at each time the patient should be well dried, and covered until another application should be made. It was also directed that an iced water injection should be given every hour or two; but three, however, were given, all of which were retained many hours, and seemed, at each administration, slightly to benefit his breathing. I continued with the patient for a while, but left him about 4 o'clock, directing that the prescription should be followed punctually, until my return, which I said would be at 7 o'clock. A suspension of the prescription, however, took place for a short time, owing to the idea that the patient must die. I returned at 7 o'clock and found him about as I had left him.

Dr. Lawson again saw him with me, and we still continued the same prescription. A slight convulsive action was observed in the flexor muscles of the left hand, accompanied with laborious breathing. Death seemed to be almost inevitable. At this moment I dashed a large quantity of water over him from a considerable height, which slightly relieved the breathing. He was now denuded of all covering, and laid upon a straw bed; iced water was then dashed on him from head to foot, and continued for some time. He was dried and covered with a blanket for a few minutes, when he was again subjected to the same process; but no good effect, further than a slight relief of the breathing, could be discovered for three hours, when I found that during the time when a full stream of water was falling on him, a general shivering took place, as though he had an ague fit. The moment this chill occurred his breathing became perfectly easy. I now felt certain that he was safe. He was dried well, and placed on a comfortable bed. Ice was still kept to his head (which had been done from the time I first saw him,) and was continued for an hour, before the termination of which time, he spoke. It should be noticed that in using the iced water, the patient was turned over each time of its application, so that it could be dashed on his back. It should also be mentioned that more than half a bushel of ice was used, with a very large amount of water—probably a barrel.

Dr. Woodward saw the patient with us during the afternoon, and agreed with us that no medicine should be given, but a dose of oil for the purpose of carrying off the calomel which had been taken, and the patient took no other medicine. He was well in a few days, and returned to his family.

In reviewing this case it may be said that the recovery was most unexpected, and may, possibly, have been in part owing to the former habits of the individual; yet it seems almost certain, that without the treatment which was adopted the case would have resulted unfavorably. As has been stated, at each application of the cold douche, the breathing was considerably relieved of its stertorous character, at least for a few minutes; though it was long before any other good effect could be observed. The chill that immediately preceded the establishment of natural breathing, was in all probability the consequence of the almost constant showering. During the three hours immediately preceding this chill, I was with the patient every moment, and used all the iced water I thought was admissible, as consistent with the continuance of life.

One thing seems to me highly probable; that an equivalent amount of opium, would have been certain to have destroyed life by apoplexy. This is one argument, I think, in favor of the use of morphine instead of opium; an argument which others have used before.

Cincinnati, Ohio, November, 1845.

